# (Un)sweetened deal? Young people’s views on the South African Health Promotion Levy and food in Khayelitsha, Cape Town

**DOI:** 10.1371/journal.pgph.0005901

**Published:** 2026-01-30

**Authors:** Hannah Graff, Namhla Sicwebu, Janet Seeley, Benjamin Hawkins, Alison Swartz

**Affiliations:** 1 Department of Global Health and Development, Faculty of Public Health and Policy, London School of Hygiene and Tropical Medicine, London, United Kingdom; 2 School of Public Health and Family Medicine, University of Cape Town, Cape Town, South Africa; 3 MRC Epidemiology Unit, University of Cambridge, Cambridge, United Kingdom; 4 St George’s School of Health and Medical Sciences, City St George’s, University of London, London, United Kingdom; PLOS: Public Library of Science, UNITED STATES OF AMERICA

## Abstract

On 1 April 2018 the South African Health Promotion Levy on sugary beverages (HPL) came into effect with the goal of lowering the consumption of sugar sweetened beverages (SSBs) across the South African population. The Republic of South Africa, following the economic and nutrition transitions that occurred after the end of Apartheid, has one of the highest rates of obesity in sub-Saharan Africa. Within this context, this qualitative study examined the food choices and food access of young people living in one neighbourhood of Khayelitsha, Cape Town in 2019 during the period soon after the implementation of the HPL. Through seven focus group discussions, we spoke with 71 young people (18–34) about their lives in Khayelitsha, their understandings and perceptions of food choices, food access, health and the HPL. Using a critical theory approach, we assessed the role of on-going income inequalities and high rates of unemployment on the lives of young, low-income South Africans, as well as how this continued to impact their food access, choices, and awareness of health. At the time of this study, the HPL was not having the desired impact in shifting and lowering the consumption of SSBs amongst this population. We used this exploration of the individual experience of the HPL in Khayelitsha as a metric for a critical policy reflection over time; adding to the small number of qualitative studies on this topic, strengthening the evidence for the inclusion of social and historical context in assessing global health interventions in local settings.

## Introduction

South Africa (SA) has one of the highest rates of obesity in sub-Saharan Africa, with levels continuing to increase [1–3]. One third of men and two thirds of women are overweight or obese. This compares globally to 43 percent of men and 44 percent of women who are overweight and 16 percent of all adults who are obese [4,5]. In SA, diabetes and heart disease are among the top five leading causes of death behind COVID-19, HIV and stroke [6]. At the time of this study the rates of overweight and obesity in children and adolescents in SA were comparable to those seen in high income, industrialized countries 10–15 years prior suggesting a continued rise in prevalence across the population [7].

Alongside its overweight and obesity, and wider noncommunicable disease (NCD) burden, SA has a declining but continued high burden of HIV. Approximately 18% of 15–49 year olds were living with HIV at the time of the study; of which 5.5% were young people aged 15–24 [8–10]. The intersection of HIV-NCD coexisting in developing economies is an evolving area of public health research, policy and practice, and the double burden of NCDs and HIV has already and will continue to impact the SA health system, economy, and communities [11,12].

South Africa’s rates of obesity and NCDs have been attributed to a nutrition transition linked to globalisation and greater freedom of movement for black populations, particularly following the end of Apartheid, and is similar to other transitioning economies in Africa [13–17]. The nutrition transition included increased consumption of food away from the home as well as increased consumption of convenience foods (e.g., pre-packaged meals), which are high in saturated and trans fats, salt, sugar, animal sourced and processed food, and sugar sweetened beverages (SSBs) [15]. Evidence suggests that consumption of sugary drinks is associated with weight gain across populations globally, including in SA [2,18,19].

Further to the nutrition transition, SA has many economic and structural similarities to other post-colonial, sub-Saharan countries, however the documented racialised income inequalities; food insecurity; brand loyalties; and particular geography, health, and cultural life in townships is a unique legacy of Apartheid policies. In 2018, 11% of the SA population was living in hunger with many more being food insecure [20]. Black South Africans are consistently in the lowest income groups [21]. There is evidence of strong brand association with regards to food choices and personal identity, particularly among lower income communities [22]. Like the global market, Coca-Cola Co., PepsiCo Inc. and the Danone Groupe have come to hold the majority of the total soft drinks sales market in SA [21–24].

When the South African Health Promotion Levy (HPL) came into effect in 2018 it had the stated goal of lowering the consumption of SSBs across the South African population. The HPL was implemented as an excise fixed rate tax on sugar content, taxing beverage products that exceed 4 grams of added sugar per 100ml [25]. At the time, this was the equivalent of an 11% levy on sugary beverages or 2.1 cents per gram; payable by the manufacturer on products to be sold domestically in SA. For the price of products to impact population diets, there is the rationale to use measures that will influence food supplies dominated by cheap, processed, energy-dense products. A primary purpose for taxing SSBs is to decrease consumption across a population, and thereby reduce diet-related risk factors for NCDs, in addition to raising awareness about SSB over consumption and health [25–27]. There are a range of SSB taxes and levies in place around the world, including a growing number in sub-Saharan Africa following on from the HPL success; evidenced by reductions in purchasing and consumption of SSBs across the population [26,28–31].

Globally, there has been a move to implement policies to address the drivers of NCDs. The last two decades has seen increased political support for the global NCD agenda evidenced by the United Nations’ high-level meetings on NCDs, in particular those in 2018 and 2025 and by the World Health Organization’s (WHO) on-going promotion of these policies. The implementation of the HPL is an example of the efforts in SA to address NCDs [25].

In the years leading up to the introduction of the HPL, 15–24 and 25–34 year olds had the two highest levels of SSB consumption in SA [32]. As well, 18 is the age at which individuals are considered to be adults in SA and may be starting to make their own decisions regarding purchasing and budgeting for food. This paper explores young peoples’ experience and perceptions of food choice, food access, the HPL and health in Khayelitsha, a township characterised by persistent inequalities, poverty and health burdens, during the period soon after implementation. It draws on a qualitative study that used focus group discussions through a critical theory approach to explore participants’ knowledge of the HPL in the context of their access to food and their everyday lives. We examine the knowledge participants had of the HPL 18 months after it came into effect and what, if any, bearing the HPL had on their day-to-day lives then. We examine the role of on-going income inequalities and high rates of unemployment on the lives of young, low-income South Africans after the implementation of the HPL. Using the HPL and taxes on SSBs as a critical lens through which to understand the interplay between culture, policy and lived experiences. In considering the impacts of policies such as the HPL, it is important to pay adequate attention to the social and historical context, and the broader realities of people’s lives. Examining the recent past to appreciate the path dependencies that extend into present and future policy debates, to avoid limiting their success. Many evaluations and examinations of fiscal policies are quantitative and this study adds to the collection of qualitative studies examining the lived experience of SSB consumption and the HPL [1,30,33–35].

## Methods

### Ethics statement

Participants were provided with written informed consent information prior to each focus group; this was provided in English and Xhosa. Oral information and facility for verbal consent was provided to enable full accessibility; all participants provided written formal consent. Consent forms included permission to use direct quotes anonymously. Interview recordings, transcripts and notes are kept confidential and stored in a password protected file on the London School of Hygiene and Tropical Medicine server.

This study received ethics approval from the London School of Hygiene and Tropical Medicine ethics committee in June 2019 (17462) and the University of Cape Town Human Research Ethics Committee in August 2019 (472/2019).

### Study setting

This was a small qualitative methods study which used focus group data collected in Khayelitsha, Cape Town. Khayelitsha is an urban township located in the Cape Flats, approximately 30km to the east of Cape Town city centre. It is densely populated and at the time of the study had one of the highest prevalence of HIV in the Western Cape. Townships are a physical legacy of racially segregationist Apartheid policies that forced migration of Black populations out of city centres [36]. Over the past 3 decades, Khayelitsha has transformed from a settlement to an established community where residents live but the majority still commute to Cape Town city centre to work [37]. There is a growing middle class population in Khayelitsha, however at the time of data collection in 2019, 89% of households were considered moderately or severely food insecure and there were high rates of unemployment [38].

### Study participants and recruitment

The study population was 18–34 year olds living in Khayelitsha. A study population of 18–34 year olds was chosen for a number of reasons. First, 18 is the age at which individuals are considered to be adults in SA and participants at this age may have been starting to make their own decisions regarding purchasing and budgeting for food, and some had begun to transition away from their family homes. Second, in the years leading up to the introduction of the HPL, 15–24 and 25–34 years olds had the two highest levels of SSB consumption in SA [32]. Finally, this age range is when the early determinants for many NCDs begin to develop, particularly those of the metabolic system such as excess weight gain.

Focus group discussions were held with community members from one neighbourhood in Khayelitsha, Cape Town in September 2019. They were organised through an established partnership between the School of Public Health and Family Medicine at the University of Cape Town (UCT) and the local community in Khayelitsha. Given the small size of the study, the recruitment of participants took place through a long-standing, established UCT field site and a local community leader. The local leader who managed the recruitment of participants is well known and respected within the community. Recruitment was conducted by advertising the study through church services, fliers, WhatsApp groups and word of mouth. Our community leader recruited broadly within the specified recruitment criteria, beyond his personal connections, and with some participants approached spontaneously as they passed by. The sampling combined purposive and convenience elements, helping to reduce potential selection bias. The focus group discussions were all held at the local leader’s church in Khayelitsha. We reflect further on our sampling and the size of the study in Limitations at the end of this paper.

isiXhosa is the primary language spoken in Khayelitsha and a co- primary investigator (PI) who is a native Xhosa speaker co-facilitated the focus group discussions and any conversations directly linked to the research; ensuring there was translation between Xhosa and English as needed. Focus group discussions were conducted in a mix of English and Xhosa. Focus group transcripts were reviewed by a third-party Xhosa speaker for accuracy.

### Data collection

Data were collected over the course of seven focus groups in September 2019. A total of 71 people participated. Each focus group was larger than is usually recommended to accommodate more participants within the temporal limitations of the study [39,40]. This allowed us to capture a wider diversity of perspectives both within and across the focus groups. Following the initial, iterative data analysis of the focus groups, occurring in parallel to the focus groups, it was deemed that data saturation had been achieved by the sixth and seventh focus groups with no new themes identified. Open-ended, semi-structured questions informed by the literature were used to guide discussions (see S1 Text).

### Data analysis

In conjunction with the data collection and following its conclusion, the focus group recordings were translated, transcribed, and coded (see S1 Table). Descriptive coding as opposed to an “in vivo” approach was used to allow the codes application across all of the data [41]. This was conducted by one of the PIs with the other reviewing to capture any thematic areas not identified. There was a discussion following each focus group to identify themes and patterns prior to formal analysis of focus group transcripts. The descriptive codes were then clustered into categories to detect patterns of frequency and interrelationships; themes were identified [41].

A critical theory approach was chosen during initial data analysis using a quasi-iterative approach, which did not impose a pre-conceived theoretical framework on the data prior to initial analysis but rather was chosen in tandem with the organising and examination of the themes identified. This allowed for the broad examination of consumers – in this case the young people we spoke to – within their setting [42,43]. We conducted an analysis of the interplay between culture, policy and lived experiences which came in the form of critically reflecting on a public policy and the idea of improving people’s lives by “shaping social structures” – the manipulation of food costs to influence purchasing and consumption and market forces driving reformulation of products – within the trajectory of the HPL to-date. For analysing our specific discussions on the HPL we assumed that fiscal policies targeting commodities are designed to potentially improve population health by four means: forcing or encouraging companies to reformulate their products; raising the price for consumers leads to a drop in purchasing and a drop in consumption; raising public awareness about the amount of sugar in the diet and its effect on health; and raising revenue from taxes earmarked to fund other health promotion [26].

### Positionality

The lead authors were both doctoral students at the time of the study. They conducted the fieldwork together. This article presents data collected for HG’s doctoral thesis. HG is a white, Anglo-American woman based in the United Kingdom, with a background in Medical Anthropology and Public Health policy development and advocacy. She has experience in a range of qualitative data collection methods in both English and non-English speaking study populations. NS is a black South African woman with training in Social Anthropology and Public Health. Although she did not grow up in a township, she shares significant cultural traditions with the participants and has extensive fieldwork experience in similar settings. This positionality helped facilitate open dialogue and deeper interpretation of cultural nuances throughout the study.

## Findings

Our findings are presented here in two primary thematic categories as follows: first our discussions on food, including the HPL as experienced at the time; and second our discussions on health. The narrative presentation of the findings brings together the key sub-themes identified, allowing for the nuanced connections between all of them Table 1. The HPL discussions are expanded on in greater detail given its role as the contextual basis for the study. The findings are expanded on in the discussion through an analysis of culture, policy and the lived experiences of the young people we spoke to as part of the wider context of the HPL.

**Table 1:** Overview of primary themes.

	Primary Themes	
	Food	Health
*Interconnected sub-themes*	Preference	Chronic disease, NCDs
	Knowledge of food, cost, nutrition	Knowledge of health, nutrition
	Cost and access	HIV
	**Impact of policy – the HPL**	Impact of policy – giving health

Participants were split approximately in half between women (n = 37) and men (n = 34) and ranged in age from 18-35. The largest sub-age groups represented were those 31–35 (n = 24) and 21–25 (n = 22). Two of the focus groups were women only and one was men only, with the other four groups mixed. All of the participants were Black South Africans. Two thirds (n = 44) of the young people in the discussions classified themselves as unemployed; thirteen said they were students; and fourteen stated they were employed (either part- or fulltime).

Participants described living in a stressful environment with low incomes and high levels of crime. This could be attributed in part to high levels of unemployment; communal xenophobia at the time towards non-South African black immigrants; and the physical location of townships such as Khayelitsha [37,44]. In addition to the stress of living in Khayelitsha, family structure was identified to be a contextual determinant of what participants ate and drank. The participants lived in one of three types of households: 1) larger, multi-generational households with one or more of income earners, where those bringing in income were the primary decision makers regarding food and budgeting, 2) single, mother-led homes, where often the sole monthly income comes from child benefit grants or 3) a handful of our participants lived independently or with one or two friends or similar aged family members in informal housing on the edges of Khayelitsha, with their income coming from employment.

### Food choice and access in the context of everyday life

This section presents the broad sub-themes identified to fall under the primary theme of Food and covers preferences, knowledge, cost and access, and the HPL. The young people of Khayelitsha were making daily choices about what they ate and drank based on a number of socio-contextual and financial factors including their preferences and the cost of products. For example, the favourite foods of the participants included “junk food” (i.e., ultra processed foods such as crisps); fat cakes (or vetkoek which are savoury fried dough buns); and sugary beverages.

The young people in our focus groups overwhelmingly expressed a liking for the sweet taste that comes from sugar. This was evident in cooking, general food consumption, and in the consumption of sugary drinks. One woman in her thirties said of her cooking:

*When I am making bread, yeah, I do [add sugar]. Sometimes if I am making, like beetroot salad. Mostly in salads and when I am making bread. And drinking tea. [Woman two in fourth focus group]*

This was followed by another woman saying:

*That’s when she puts lots and lots of sugar [in] (with laughter from the group). [Woman three in fourth focus group]*

The amount of sugar added to food and drink came up throughout the conversations. There was a social awareness of excess sugar, echoed by many, for example here by one man in his teens telling us how he takes his coffee:

*And when you drink coffee, you take a big spoon, one, two, it must be sweet. Like, if you ask me how many spoons for you, I will say two. But then I sneak more later. [Man two in fifth focus group]*

A large portion of the sugar these young people consumed came from sugary drinks. Often sugary drinks were a choice over other options, in particular water. Across the groups, we were told about all of the different types of sugary drinks people chose. This list includes “Twizza and Jive”, and “Fusion” (a concentrated juice), and “Coke”. We were told about making “sweet water” by just mixing sugar and water and about having a fizzy drink every day after dinner, usually a “Jive mos”.

When asked what people drink in Khayelitsha besides fizzy, sugary drinks many of the young people mentioned juice as being the next option. However, juice was deemed as often being too expensive for regular purchase. It was noted that if people wanted juice, people could often only afford concentrated fruit juice or squash, which is then diluted with water. The other particular point about fruit juice, which was made in several discussions, was how fruit juice was not “acidic” enough, and that people in Khayelitsha prefer to buy drinks that have acid – fizzy drinks – but also those drinks were less expensive than juice or bottled water. When talking about the choice of fizzy, sugary drinks in particular, the discussion often came back to “Coke” specifically by name and the acknowledgement that Coke was more expensive than other options. Brand loyalty to Coke in relation to the HPL and socio-economic realities are explored in our discussion.

When choosing foods and drinks, young people felt their parents did not teach them about making food choices or healthy foods:

*…it’s rare to find parents who tell you to eat healthy foods - there is [sic] only a handful of parents that do that… [Woman one in first focus group]*

Similar sentiments were felt by a young woman in her teens, saying:

*So, these breadwinners, are people from the past, older people who know nothing about food and this person works, so obviously, he will want to eat nice every day. What is nice to a black person? Meat and fast food. So, I don’t think breadwinners eat healthy, because they know nothing about health. The people that actually have some level of health knowledge are us, because we are at least taught at school and everything. [Woman one in fifth focus group]*

In addition to these discussions about parents – and others within their households – as a source of mistrusted knowledge, there were also several conversations regarding where people learn about food and health in Khayelitsha. In these discussions the link between food and health was not just nutrition. The nutritional value of foods was only a small part of how these young people linked food with their health, and their food choices; taste, cost, brand association and family influence were all determinants. For example:

*We don’t have a choice of what we eat… From the small shops that you find around the corner, what you only find here is the same thing. If you ever decide that you are going to do yourself a sandwich, let’s say you are going to buy a bread, you are gonna buy cheese, tomato and eggs and what you call? Avocado…that thing is like too much. But if you go around the corner, you will find a fat cake there. [Man three in second focus group.]*

This is a useful illustration of the accessibility – physically and financially – of food and the knock-on effect of what people then choose to eat. Overall, the young people in Khayelitsha said food was generally expensive. For example, one woman in her twenties said:

*It’s very expensive. When you go make [sic] grocery you must look ‘Oh this one is cheaper than this one’ you must compare prices so that you can get the lesser one than the other one. [Woman five in fourth focus group]*

And further to this, when we asked in the first group about buying food, a man in his twenties simply said, “The problem is money.” In the discussion, the role of food in young people’s lives and health is presented as a mix of choice and access within the interplay of culture, policy and lived experiences.

Money and the price of food, as just presented, as well as who made decisions about food purchasing and when, were woven into all of the focus group discussions. When discussing income and the price of food, and when asked about the HPL directly, the majority of participants said they had noted the change in price in sugary beverages over the preceding 18 months, however they did not know why the price had changed; many only learning about the levy through our discussions. For example:

*No, I just noticed the change in prices. They were cheaper before, but now they are expensive. [Woman five in third focus group]*

And:

*No [we did not know about it], but we can see that things that contain sugar are more expensive. [Man two in first focus group]*

This lack of awareness of the HPL led to talking about what the levy was and what it was meant to do. We discussed the implementation of a tax such as the HPL and participants shared their thoughts on why the government implemented it. For example:

*Somehow, I think they made the sugar tax applicable to us because there are many diseases or sicknesses that have been going around, so apparently the Department of Health has requested for sugar tax. That’s why fizzy drinks, the prices have risen…. [Man two in seventh focus group]*

As well as whether it might work, participants held a generally negative outlook about the effect of the levy on changing consumption patterns in Khayelitsha.

*No [it is not going to work] (laughter). Not by a long shot. [Woman two in fourth focus group]*

And pessimistic as to the government’s motivations:

*I think the sugar tax is just there to disadvantage the lower-class people because even if the Coke is R30, the middle- and high-class people can still afford it but lower-class can’t, so. [Man one in fifth focus group]*

These sentiments, that the tax was not going to work, parallel what we were told regarding sugary drink preferences; analysed in context in the discussion. We were told how budgets were managed and manipulated to allow for even the occasional purchase of name-brand SSBs despite price rises. These were items the young people were not willing to give-up:

*I never stop buying Coke. [Man two in fifth focus group]*

### The experience of health in Khayelitsha

Throughout our conversations the presence of chronic diseases and NCDs amongst the community was obvious. Many of the young people knew people or had older relatives living with diabetes, heart disease and cancer; some dying from these conditions. And an acknowledgement of the potential harm from fizzy drinks and how they “create” conditions like diabetes. Multiple participants indicated that managing chronic diseases in their families was stressful and had direct impacts on their daily lives.

In talking about chronic disease more broadly, beyond their immediate household experiences, the participants had a wide range of commentaries. For example, there were explicit links made between sugar and chronic disease. One woman in her twenties said:

*I will say for me it's sugar that I am worried about because they say it causes gallstones, and the problem is you never know when its growing [Laughter]. Having diabetes is also an issue. [Woman eight in fourth focus group]*

In talking about chronic disease and if these young people thought about their long term health, there were mixed responses. Some said they did broadly think about “being healthy” and avoiding “acid” and others were more specific. For example, a young man in his twenties told us he did think about his long-term health and said this included:

*Like sugar diabetes…hypertension…all those kinds of diseases…stroke…high blood pressure…those chronic diseases. [Man three in seventh focus group]*

While others said they did not think about their long-term health. For example, a man in focus group five told us:

*It’s because we don’t know tomorrow and that’s why sometimes when you have R30, you think of buying healthy stuff, but then again think, I might even die today or tomorrow, it doesn’t depend on what I eat. [Man six in fifth focus group]*

When speaking about the context of their health more broadly, the presence of HIV within the community also came up in the discussions. For example, a man in our last group said:

*…they are the ones that are in front now…diabetes and HIV*. *[Man five in seventh focus group]*

This illustrates the double burden facing SA. There was also this observation, by a different man in his twenties in the same group:

*HIV and the diabetes are two different things. You can live long when you got HIV, but you can’t [live] long when you’ve got diabetes. [Man one in seventh focus group]*

In the discussion, we unpack in the context of township life, how this sentiment conveys that HIV had become chronic and something people in this community live with, whereas diabetes was new and having an acute impact.

These discussions around HIV in the community and its impact on the broader health context were intertwined with the discussions on sugar and policy. For example, a young man in one of the first discussions explained how he thought the government should “produce health” in the same way they had promoted the HIV prevention response:

*About um the government supporting the health thing, the health environment by the tax... I would like them, as it is they are producing help for people to use condoms, for prevention, they should use the same system in producing health… The government should also give out, as they are giving out free contraceptives and everything like, hand out to people like, like the old people, give them their own type of grocery bag. [Man five in first focus group]*

A further example from a woman in the first group:

*If there was a system like that…like you don’t pay for your health, you go and get your health. Then everyone can afford to buy what they need extra for their own health, so the whole family will be secured and safe. [Woman eight in first focus group]*

This idea of the government “giving” or “producing” health appeared in different forms and wording across the groups; and is discussed later as part of our analysis. As part of the idea of the government “giving health”, the young people in Khayelitsha suggested that more could be done to increase individual’s knowledge about food, and food’s impact on health. In the Discussion we expand on how the SA government’s response to HIV and post-Apartheid legacy in-part shaped the broader health landscape, setting up the context for the HPL to sit in Khayelitsha at the time [44].

## Discussion

This study used SSB taxes as a lens to explore young people’s immediate, lived experiences; examining how price changes and policy influenced food choices and health knowledge during the period soon after the implementation of the HPL. It offers a critical analysis of culture, policy and lived experiences within the expanded context of the HPL today and the assumed design of fiscal policies targeting commodities, as outlined in Methods. This analysis is placed here within our discussions with young people – consumers in their setting – providing an example of qualitative fiscal policy assessment and insight of a global health intervention in a local SA setting.

The primary argument against the implementation of SSB taxes is that they are by design economically regressive; in that lower income households pay a larger proportion of their income with any additional tax added to a product as compared to higher income households [26,45,46]. The global health community advocates for these policies, with real-world and modelled evidence supporting their use, to shift whole population consumption levels; with lower income groups, that usually have the highest health burdens most significantly impacted overtime [26]. An argument that the poorest will face the largest financial burden of such policies is often used to fight their implementation [26]. Since HPL implementation, several studies have found a decrease in purchasing and consumption in low-income and high-consuming sub-populations in SA.

Reduced purchasing is corroborated by our findings and many of the young people noted the rise in price of Coke and other brand name sugary beverages. Our participants spoke of a shift in purchasing based on the change in price in sugary beverages – even if many of the young people did not know why the price had changed. However, our results show that the shift was most often to less expensive, non-name brand sugary drinks or to purchasing at different times in their pay cycle or budgeting to afford Coke. This is as opposed to making a shift to lower or no sugar, healthier drink options altogether. This is consistent with evaluations of the HPL conducted after our study that found shifts to no-tax sugary beverages [47,48]. This illustrates an initial, acute economic impact of the HPL on this population that had a noticeable and immediate effect on low-income purchasing behaviour at the time.

While the HPL impacted behaviour amongst the young people in Khayelitsha, at the time of this study a large proportion of the participants indicated they had not heard about the HPL until our discussions. This is consistent with the literature and discussions with our local gatekeepers [1,49]. For example, Bosire et al. (2020) investigated perceptions and attitudes among urban South Africans who were living in Soweto on factors that contributed to their SSB intake as well as South Africa’s use of a tax to reduce SSB consumption in the three months prior to the HPL implementation in 2018 [1]. Participants in that study reported frequent SSB consumption and attributed this to habit, addiction, advertising and the wide accessibility of SSBs. Regarding the levy itself, most participants were not aware of the proposed tax and indicated cynicism with regards to the government’s stated motivation in introducing the tax [1]. Studies since our data were collected suggest that even with changes in purchasing and consumption, general knowledge of the HPL has remained low; or even decreased over time [33].

Alongside the literature on knowledge and awareness of the HPL, there are mathematical simulations and modelling studies which were conducted to estimate the potential changes in consumption and related health outcomes prior to the HPL implementation [2,32,50,51]. For example, Manyema et al. (2014) estimated the impact a 20% tax on sugary drinks might have on obesity rates in SA, constructed on estimated consumption changes based on price elasticities and thus calories consumed based on those estimated changes in consumption [32]. Following the HPL implementation, further quantitative evaluations indicate actual decreases in SSB intake [31,52].

As part of critically understanding the context and immediate experience of the HPL, in our discussions both on the health context of Khayelitsha and on the HPL, a noticeable majority of participants indicated they already knew that drinking sugary drinks was not good for their long-term health in some way. This is consistent with the findings of other studies published since HPL implementation [33,47]. This knowledge regarding the un-healthiness of sugary drinks was not linked to any prior knowledge of the HPL. In thinking critically about the role of policies on health, there is historical evidence to support the argument that structural violence – in this case high levels of unemployment and post-Apartheid structural and geographic legacies specific to SA – is a determinant of health [53]. This study, which has identified these wider determinants, adds to the small collection of qualitative studies examining the lived experience of SSB consumption and the HPL, and of SSBs more broadly within a local setting.

As highlighted earlier, the strong brand association, loyalty, and conspicuous consumption seen in our discussions is evidence of the financial and physical legacies of Apartheid, particularly in relation to Coke versus other local fizzy drink brands. We know Coke and the other leading global fizzy and sugary drinks brands have held the majority of the market for many years [22]. This market control – and the social aspiration and status associated with being able to drink Coke – were evident in our discussions. Participants told us that Coke was their preferred beverage and it was also evident in the sheer presence Coke had within the physical environment; Coca-Cola’s bright red signage and distinctive white font was everywhere. The pricing structure of Coke products in SA may have shifted because of the HPL, but at the time of our study the influence of Coke within Khayelitsha had not [54].

Post-Apartheid structural legacies, as previously mentioned, in particular SA’s racialised income inequality and life in townships influence diet, obesity rates and responses to policy. Our participants – who were all Black South Africans, and largely unemployed – reflected the high levels of poverty in SA and is consistent with Black South Africans being consistently in the lowest income groups [21]. Much of this perpetuation of poverty is driven by the geographic and physical legacy of townships. Note for example the distance Khayelitsha sits from the Cape Town city centre. As well as education and employment opportunities, there is evidence globally to support the idea that individuals’ food choice and diet is associated with levels of unemployment and poverty [55].

Furthermore, food insecurity is well-documented in townships, with evidence aligning with our results that people lacked access to food at specific times of the month depending on when they were paid or received benefits, and were often unable to consume the foods they would prefer or knew to be healthy [24,44,56–58]. Further evidence suggests that additional factors influence purchasing and consumption beyond explicit cost impacts from the HPL, such as the prices and availability of products at small retailers (i.e., spazas and tuck shops) in a low-income township [48]. These shops will have been managing changes in price too, passed on from manufacturers. In addition, consumers lack clarity in the use of tax revenue raised by the HPL [35].

The idea that macro-level social, political and economic forces shape individuals’ health, in particular the impact of policy, social structures and individual and community experiences of health, facilitate a critical assessment of lived policy [43,59]. The HPL is by design meant to shape one element of people’s food environments in an effort to reduce their levels of sugar consumption. However, other large socio-cultural structures identified through our discussions were shaping these young people’s lives at the time. Including, but not limited to, the post-Apartheid structural legacies of continued economic and geographic barriers. All paired with an interplay of old (e.g., HIV) and new (e.g., NCD) health realities.

Further to the critical assessment of consumers in their lived realities – and old and new health realities – is the impact of COVID-19 [60,61]. Evidence suggests that, since the start of the pandemic in 2020, responses to COVID-19 have impacted the control, prevention and diagnosis capacity of both HIV and NCDs [61]. Hypertension, diabetes and HIV were the leading co-morbidities of COVID-19 mortality in SA; and the pandemic exacerbated malnutrition, particularly in low-income settings such as townships, because of lockdowns and the broader global response [62–67].

The lived realities in parallel to government responses to old, new and emerging health realities were apparent at multiple points throughout our focus group discussions. Primarily when speaking about the health context of Khayelitsha and scope for policy change, the idea of “giving” or “producing” health was discussed; as a right and expectation. It came up in talking about the legacy of HIV and the SA government’s programs to promote prevention by “giving” out condoms and therefore “producing” better health. It came up when talking about the HPL itself and how the government should not tax those already living on low-incomes, but rather “give” them healthy foods through subsidisation. It also came up when discussing what the government could do to help their health beyond the HPL; they could “give” them more jobs. The “right to health” is advocated by the WHO and enshrined by the United Nations, and our findings reveal that the young people in Khayelitsha had an overwhelmingly collectivist understanding of their government’s responsibility to uphold this right [44,68]. SA developed a comprehensive response, over decades, to the HIV pandemic, however broader structural barriers (e.g., political, economic, socio-cultural) were limiting the HPL in this setting from “giving” young people a healthier food environment for which to make individual decisions.

By focusing on the interplay of culture, policy and lived experiences we examined the role of policy in shaping lives, and health, beyond the global public health discourse and accepted policy mechanisms. This is as opposed to a systematic “policy analysis” which might undertake a stakeholder analysis; or use a specific theoretical structure for policy development and agenda setting; or critique the political dynamics of a specific public policy within a set framework [69–71]. This was a point in time study and a direct or indirect response from the SA government was not included in the study design. However, it can be used to inform future policy refinement and development.

Elements of the policy mechanism that emerged from our analysis – primarily price shifting consumption, public awareness, and revenue for public use – did not fully align in Khayelitsha in the first years after the HPL implementation. While some evaluations to-date do point to shifting consumption and the population impact from the HPL, including among low income consumers, and the overall success of the HPL [27–29,31,34,47]. Our study supports others’ findings that lived realities and expectations of the HPL are not as simple as reductions in purchasing.

No population level policy is entirely equitable across the population, however using qualitative data such as ours from early in the policy’s tenure alongside evaluations overtime should be used to enhance the effectiveness of the HPL and equity issues by addressing, for example, public awareness and wider structural determinants.

### Limitations

There were a number of limitations and challenges to this study, both practical and temporal. This was a small study conducted in one neighbourhood and this limitation on size was mitigated through on-going refinement and reflection of the aim, objectives, methods and theoretical framework to keep them simple. However, the actual number of participants was slightly higher than anticipated, as explained in Methods, and therefore a substantial set of data were collected. There was a potential for selection bias amongst our participants as they were all drawn from the same areas of Khayelitsha. However, recruitment was conducted within the specified recruitment criteria and our sample combined purposive and convenience elements. We were also limited by when we were feasibly and safely able to conduct the focus group discussions. It would have been unrealistic and unsafe for us to conduct focus group discussions in the evenings and therefore they were all held on weekdays in the middle of the day. Therefore, we were always likely to get higher than average numbers of participants who were unemployed, which will have impacted our results. The final practical limitation to this study was the fact that one of the PIs is not a native Xhosa speaker. As discussed in the Methods, this was mitigated by having a native Xhosa speaker as a co-PI and facilitator of the focus group discussions, as well as having a third-party Xhosa speaker review the translated transcripts against the discussion recordings for accuracy.

In addition to these practical limitations, there is a notable temporal challenge to this study. The timing of the data collection, conducted in 2019, in relation to when the HPL was implemented in April 2018 and subsequent changes in the policy landscape. This is a limitation with regards to the other evaluations and assessments of the HPL conducted at the time of data analysis for comparison and in the intervening period in which our discussion was developed; in addition to disruptions caused by COVID-19. It is also a limitation in that population level policy measures such as the HPL are designed to have an impact over a number of years, or decades, before they yield change in behaviour or health outcomes at the population level. This was mitigated through the study design. It was never the intention of this study to examine long-term health impacts of the HPL on young adults in Khayelitsha, or to be a direct policy analysis for feedback to government, but rather to highlight day-to-day experiences of the HPL at a point in time.

## Conclusions

This article utilised data from focus group discussions to reflect critically on the HPL’s impact on the day-to-day lives of young people during the period soon after implementation, within the larger context with which it sits. It highlights the role of on-going income inequalities and high rates of unemployment on the lives of young, low-income South Africans at the time. In Khayelitsha, the HPL had yet to impact levels of consumption but had impacted young people financially. This article adds to the literature on structural barriers to health (i.e., racialised unemployment); the broad impact of inequalities on health (i.e., a socio-economic environment limiting healthy food and drink consumption); and the right to health (i.e., government subsidised prevention interventions) [42,43,72]. This study also moves the literature forward by examining the effects of a policy intervention which is popular amongst many public health advocates and policymakers, and is designed to shift some of the health burdens seen across populations. Understanding the recent past of a policy’s life span is important for appreciating the path dependencies that extend into present and future policy debates. Our findings highlight the historical and contextual picture of the HPL success, as well as broader global health interventions in local settings, and provides additional contextual information for future evaluations and policy developments.

The SA government stated a desire to address widespread inequalities and the rising rates of obesity and other NCD risk factors in the country [73]. NCDs sit within a health and social context that has been dominated by the response to HIV and post-Apartheid legacy for over 30 years; and now the aftermath of COVID-19. The SA government’s implementation of the HPL was a population level policy decision supported by public health evidence and seen as a successful model for similar policies across the continent [29]. It is important for future research to examine with attention the social and historical context in which policies like the HPL are implemented and evaluated.

## Supporting information

S1 Table Data analysis framework. Data coding framework (nodes).
(DOCX)

S1 Text Topic guide. Focus group topic guide.
(DOCX)
