# Supplementary material for: (Un)sweetened deal? Young people’s views on the South African Health Promotion Levy and food in Khayelitsha, Cape Town
Source: PLOS Glob Public Health. 2026 Jan 30;6(1):e0005901. doi: 10.1371/journal.pgph.0005901 (PMC12857988; doi:10.1371/journal.pgph.0005901)
Supplement: S1 Table — Data coding framework (nodes). (DOCX) [file pgph.0005901.s001.docx]

- 1. S1 Table. Data Analysis Framework

## Data coding framework (nodes)

| Analysis nodes | Descriptions | Occurrence in a focus group | References |
| --- | --- | --- | --- |
| 1. Life in Town Two Codes |  | 7 | 139 |
| 1.A. Unemployment | Any mention of lack of jobs; no jobs; high unemployment; etc. | 7 | 24 |
| 1.B. Poverty | Mention of being poor, living in poverty, not having much, etc. | 5 | 13 |
| 1.C. Crime | Any mention of crime, violence, theft, muggings, assaults, etc. | 5 | 14 |
| 1.D. Family | Family structure; who lives in a household | 4 | 15 |
| 1.E. Foreigners | Any mention of "foreigners"; Somalians | 4 | 15 |
| 1.F. Drugs & alcohol | Any mention of drug or alcohol use in connection to "life in Town Two" | 4 | 5 |
| 1.G. Built environment | Any mention of the physical environment, make-up, lay-out, etc. of Town Two. | 2 | 4 |
| 1.H. Money & budgeting | General reference to money (separate from “poverty”) and/or the need to budget money, not necessarily just for food. | 5 | 20 |
| 1.H.1. Cost of utilities | Explicit mention of the cost of electricity, gas, water, etc. | 1 | 2 |
| 1.I. Children | A mention of children - having them, raising them, feeding them, etc. in Town Two. | 5 | 14 |
| 1.J. Local governance | Mention of the local government, governance structure, counselors, etc. in Town Two. | 1 | 5 |
| 1.K. Jobs | Any mention of a job, employment, career, etc. | 1 | 3 |
| 2. Food Codes |  | 7 | 318 |
| 2.A. Cost | Price of food; cost of food; change in the price or cost of food | 7 | 93 |
| 2.B. Preference | Choice of food; foods they like; foods they don't like; what do they eat and drink a lot of? A little of? | 7 | 73 |
| 2.C. Decision | How do they decide what food they buy? Eat? Who in their household makes the decision(s) about the food that is purchased, cook and eaten? | 7 | 58 |
| 2.C.1. Staples | Specific mention of “staple” or initial food items purchased each month in-line with budget and money available. | 4 | 8 |
| 2.D. Source | Where does your food come from? Where do you buy food? | 4 | 12 |
| 2.E. Knowledge | What do you know about food? Where do you/have you learned about food? | 6 | 21 |
| 2.F. Access | Access to and accessibility of food items. | 7 | 38 |
| 2.G. Consume | Foods eaten or consumed - may be paired with preference and/or decision. | 5 | 18 |
| 2.H. Farming | Specific mention of farming as it relates to food | 0 | 0 |
| 2.H.1. At home farming | Growing/or not growing any of your own food | 2 | 3 |
| 2.I. Cooking | Any discussion or mention of cooking, type of cooking, means of cooking, etc. | 1 | 1 |
| 2.J. Culture | Explicit reference to “food culture” or “culture” as it relates to food knowledge, choice, preference, health, etc. | 2 | 4 |
| 3. Sugar Codes |  | 7 | 74 |
| 3.A. Health | Sugar and links to health (positive or negative) | 1 | 2 |
| 3.B. Sugary drinks | Any mention of sugary drinks (fizzy drinks, soda, Coke, Twizza, etc.) - Which ones do you drink? How much do you drink? How much does is cost? Preferences? Sugary drinks and your health? | 7 | 34 |
| 3.B.1. Acid | Mention of “acid” “acidity” etc. in relation to the healthiness of sugary drinks (ie Coke). | 4 | 6 |
| 3.B.2. Coke | Specific mention of Coke, Coca-cola, etc. | 6 | 27 |
| 3.B.3. Quantity | Quantity of sugary drinks consumed | 3 | 5 |
| 3.B.4. Twizza | Explicit mention of Twizza - lower cost cola/soda drink (local to SA) | 2 | 4 |
| 3.C. Water | Any mention of water with reference to drinking | 6 | 10 |
| 3.D. General-Miscellaneous | Any additional or miscellaneous mention of sugar not related to 3A-C | 1 | 5 |
| 3.E. Cost | Cost of sugar | 6 | 14 |
| 3.F. Sweetness or flavor | Explicit mention of the sweetness or sweet flavor of something - may be paired with preference and/or other sugar codes. | 3 | 9 |
| 4. Health Codes |  | 7 | 111 |
| 4.A. NCDs | Any mention of a health condition categorized (by public health/policy) as a non-communicable disease | 3 | 6 |
| 4.A.2. Diabetes | Type I or II | 5 | 21 |
| 4.A.3. Cancer | Any specific mention of cancer - any type | 1 | 1 |
| 4.A.4. Heart disease | Any specific mention of heart disease - any type and including heart attack | 1 | 1 |
| 4.A.F. High blood pressure | Any mention of high blood pressure | 1 | 2 |
| 4.B. HIV | Any mention of HIV - including historical, cultural, legacy reference | 2 | 10 |
| 4.C. TB | Any explicit mention of TB | 1 | 1 |
| 4.D. Safety | Any mention of health equating to the safety and make-up of foods and drinks ingested… | 2 | 6 |
| 4.D.1. Hygiene | Any mention of discussion of the hygiene or cleanliness of food, ingredients or food production | 1 | 1 |
| 4.D.2. Ingredients | Specific mention of the ingredients in certain foods/food products as it links to that items safety or healthy-ness | 2 | 3 |
| 4.D.3. Production | Of food and drink - only linked with safety - and equating that to health. | 2 | 2 |
| 4.E. Food choice | Food choice linked with health; wellbeing; how does it make you feel | 7 | 32 |
| 4.E.1. Personal choice | Specific mention of personal choice vs. access with regards to food choices/what people consume | 3 | 7 |
| 4.E.2. Habit | Habit as a driver of food choice | 1 | 1 |
| 4.F. Knowledge | Where do you learn about health and/or own health knowledge | 6 | 36 |
| 4.G. Health professional | Mention or discussion of interaction with a health professional (dr, nurse, CHW, etc.) | 3 | 8 |
| 4.H. Health care setting | Health care facilities, settings, capacty | 2 | 3 |
| 4.I. Exercise | Any mention of exercise, sport, etc. | 2 | 4 |
| 4.J. Obesity-weight | Any mention of obesity, weight, size, body shape | 1 | 1 |
| 4.K. Stress | Any mention of “stress” | 1 | 4 |
| 5. Policy & the Government Codes |  | 7 | 108 |
| 5.A. Sugary Beverages Levy | Any mention of the SA sugary beverages levy | 7 | 28 |
| 5.A.1. Public Awareness | Public awareness before or after the implementation of the sugary beverages levy | 7 | 19 |
| 5.A.2. Recommendations | Specific recommendations for the funds raised from the sugary beverages levy | 2 | 6 |
| 5.B. Messaging | Mention/discussion of health messaging from the government, health professionals, food companies, etc. | 4 | 6 |
| 5.C. Interventions | Mention/discussion of what the government, health professionals, etc. should be doing to help people's health | 7 | 24 |
| 5.D. Information-education | Any mention of the information received regarding health, new taxes/laws/policy, etc. | 3 | 5 |
| 5.D.1. Social media | Mention of social media as linked to information and public awareness from government (& NGOs?) - specifically regarding health (??) | 2 | 2 |
| 5.D.2. Food company marketing | Any mention of a food company marketing their products | 2 | 3 |
| 5.E. Recommendations | Respondents recommendations to government - general | 7 | 36 |
| 5.F. Geopolitical-economic | Wider national and global political and economic factors such as the price of raw commodities, inflation, etc. | 3 | 6 |
| 5.G. Taxation (general) | Any mention of tax in a general sense, not the SA sugary beverages levy specifically | 2 | 3 |
| 5.G.1. Alcohol | Mention of alcohol taxation | 2 | 2 |
