# Supplementary material for: (Un)sweetened deal? Young people’s views on the South African Health Promotion Levy and food in Khayelitsha, Cape Town
Source: PLOS Glob Public Health. 2026 Jan 30;6(1):e0005901. doi: 10.1371/journal.pgph.0005901 (PMC12857988; doi:10.1371/journal.pgph.0005901)
Supplement: S1 Text — Focus group topic guide. (DOCX) [file pgph.0005901.s002.docx]

S1 **Topic Guide.** Focus group topic guide for investigators.

***Focus group topic guide for investigators:***

*September 2019*

What do people in Town Two normally eat?

Can you tell me/us about what you like to eat and drink?

Can you tell me/us about where you buy food and where you eat your meals?

Who in your households make decisions about meals and what foods you have at home?

Do you think about how much food and drink cost?

How do you feel when you eat?

Where did you learn about food?

Where do you learn about your health and your body?

*Interested in (not to be used as leading questions):*

Food & food access

Do you think about the food you buy / what type of food do you buy and why?

Do you find it hard to find food?

**Do you find it hard to afford food?**

Health

**Impact of sugar on overall health, weight gain?**

Do you think about the food you eat being linked to your health?

**Do you notice health campaigns or messaging?**

**Do health messages change your behaviour?**

The tax

Do you drink soft drinks/fizzy drinks?

**Do you know there is a tax on fizzy drinks that started in 2018?**

**What do you think about the tax?**

**Do you know why the government started the tax?**
